# Biomarkers-based personalized follow-up in chronic heart failure improves patient’s outcomes and reduces care associate cost

**DOI:** 10.1186/s12955-021-01779-9

**Published:** 2021-05-08

**Authors:** Antonio Leon-Justel, Jose I. Morgado Garcia-Polavieja, Ana Isabel Alvarez-Rios, Francisco Jose Caro Fernandez, Pedro Agustin Pajaro Merino, Elena Galvez Rios, Ignacio Vazquez-Rico, Jose Francisco Diaz Fernandez

**Affiliations:** 1 Macarena University Hospital, Dr. Fedriani nº3, 41009 Seville, Spain; 2 Juan Ramon Jimenez University Hospital, Huelva, Spain; 3 Virgen del Rocio University Hospital, Seville, Spain

**Keywords:** Heart failure, Personalized medicine, Patient value, Patient outcomes, Budget impact, Biomarkers

## Abstract

**Background:**

Heart failure (HF) is a major and growing medical and economic problem, with high prevalence and incidence rates worldwide. Cardiac Biomarker is emerging as a novel tool for improving management of patients with HF with a reduced left ventricular ejection fraction (HFrEF).

**Methods:**

This is a before and after interventional study, that assesses the impact of a personalized follow-up procedure for HF on patient’s outcomes and care associated cost, based on a clinical model of risk stratification and personalized management according to that risk. A total of 192 patients were enrolled and studied before the intervention and again after the intervention. The primary objective was the rate of readmissions, due to a HF. Secondary outcome compared the rate of ED visits and quality of life improvement assessed by the number of patients who had reduced NYHA score. A cost-analysis was also performed on these data.

**Results:**

Admission rates significantly decreased by 19.8% after the intervention (from 30.2 to 10.4), the total hospital admissions were reduced by 32 (from 78 to 46) and the total length of stay was reduced by 7 days (from 15 to 9 days). The rate of ED visits was reduced by 44% (from 64 to 20). Thirty-one percent of patients had an improved functional class score after the intervention, whereas only 7.8% got worse. The overall cost saving associated with the intervention was € 72,769 per patient (from € 201,189 to € 128,420) and €139,717.65 for the whole group over 1 year.

**Conclusions:**

A personalized follow-up of HF patients led to important outcome benefits and resulted in cost savings, mainly due to the reduction of patient hospitalization readmissions and a significant reduction of care-associated costs, suggesting that greater attention should be given to this high-risk cohort to minimize the risk of hospitalization readmissions.

**Supplementary Information:**

The online version contains supplementary material available at 10.1186/s12955-021-01779-9.

## Introduction

Heart failure (HF) is a major and growing medical and economic problem, with high prevalence and incidence rates worldwide [1]. It has been estimated that up to 2% of the adult population under 75-years, and up to 7.5% in 75–84 years old suffer HF, affecting more than 26 million people around the world [2]. It is a chronic debilitating illness in which the symptoms worsen with progression of the disease. Disease progression is associated with significant impact to the physical and social wellbeing, increased hospitalization as well as increased mortality [3]. Heart failure poses a significant burden to the health budgets globally. It is estimated that 1–2% of total healthcare expenditures in Europe and North America is spent for the treatment of HF. The economic burden of HF is estimated at US$108 billion per annum [4].

Primarily due to significant treatment advancements to prevent previously fatal acute cardiac events [5], the burden of heart failure characterized by chronic symptoms, acute hospitalizations and high care associate cost, continues to rise. HFrEF is an important cause of hospital admissions and the reason for more than 5% of medical hospitalizations in adults [6]. Hospital admissions account for the largest part of health costs related to HF because hospital stays are usually lengthy and become progressively more frequent. On the other hand, people affected by HFrEF experience different physical and mental complications due to the chronic and prolonged disease course which have a serious and negative impact on their quality of life [7]. Poorer quality of life correlates with increased hospitalization times and mortality rates, and higher costs imposed on health systems, families, and patients. The focal point going forward should be to maximize function in everyday life and quality of life in order to reduce the burden of care in HFrEF [8].

Despite the improvement achieved during the last decades, optimizing management of HFrEF remains a challenge. Cardiac biomarkers are emerging as a novel tool improving HF management. Personalized management of HFrEF based on biomarkers could help to address some of the challenges [9]. Recent guideline updates also give recommendations for the use of B-type natriuretic amino-terminal propeptide (NT-proBNP) for assessing the risk for hospitalization and identifying unaffected patients at risk for incident of HF [10]. However, whether biomarkers can or should be used for guiding management in patients with chronic HFrEF remains in limbo [11]. Although cardiac biomarkers guided management studies suggest significant improvement in patient mortality over usual care [12], there is not a clear recommendation regarding the precise timing and extent of contact to cost-effectively improve health outcomes. In addition, these studies did not explore end-point related with quality of life, particularly given an increasingly older and more clinically complex patient population who demand more from limited healthcare resources [13]. These clinical gaps cannot be ignored. More research to improve HFrEF management is needed in real world clinical practice as well as its impact on patient outcomes, patient’s quality of life and care associated cost. More importantly, to assist the clinicians less well versed with the guidelines who may be less familiar with managing HFrEF [14].

We performed this study to address potential uncertainties about the best approach for HFrEF management. Our main objective was to study and validate a new, real-world clinical practice approach for HF personalized follow-up based on cardiac biomarkers compared with regular care in our clinical setting. Secondly, evaluate the impact on reduction of hospitalization readmission rates, reduction in the rate of visits to the Emergency Department (ED), and improvements in patient’s quality of life assessed by New York Heart Association (NYHA) Functional Classification scores. We also aimed to evaluate the effect of the intervention on HF care-associated costs in terms of cost-savings.

## Methods

### Design and setting

This is a before and after interventional study. The design involves evaluating the effects of an intervention—new approach for personalized follow-up based on biomarkers risk stratification—in chronic HFrEF patients by comparing the outcomes, quality of life and care associated cost of study participants investigated before the intervention with those measured afterwards. It was conducted from the perspective of the Spanish healthcare system in a single academic center (Huelva University Hospital, Huelva, Spain), a 600-bed academic teaching hospital and tertiary care referral center, with all major clinical services. The Heart Failure Unit (HFU) is the referral unit for a population of 550,000 and sees approximately 1000 patients per year. The protocol was approved by the Institutional Review Board, and a waiver of the requirement for a written consent from all participants was approved.

### Participants and protocols

**Fig. 1:**
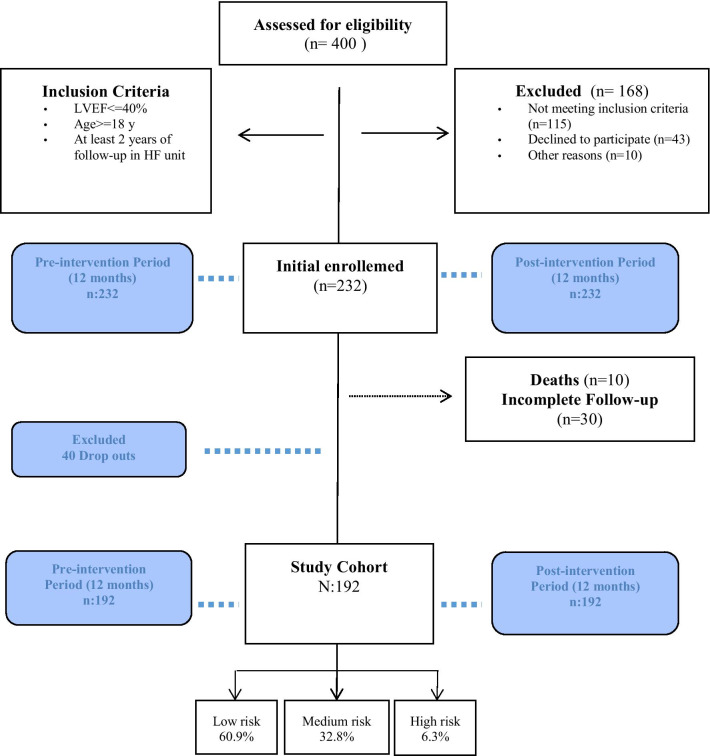
Flow-chart of included patients

The population of the study included chronic HF patients, aged 18 years or over, with a reduced left ventricular ejection fraction (LVEF) of 40% or less and more than 2 years of follow-up in the HF outpatient’s clinic. Diagnosis HFrEF was in accordance with international guidelines [10, 15]. A total of 232 patients were enrolled between June 2017 to 2018 (Fig. 1). All the patients that did not complete the post-intervention period (death or drop out) were eliminated from the analysis, in both pre- and post-intervention periods.

Non-probabilistic sampling method was used. Patients from the HF outpatient’s clinic, after verification of the eligibility criteria, were included consecutively into the study. The sampling process came to an end when the sample size was reached. The recruitment was performed by the cardiologists of the HFU.

Our Study is a single arm study with one group measured before the intervention and again after the intervention. The study was divided into pre- and post-intervention period. The pre-intervention period was defined as the period of 12 months prior to the intervention implementation. During this period, patient management followed basic care protocol based on well-articulated clinical practice guidelines and consensus documents regardless of biomarker results. All patients were visited every 3 to 6 months according with their symptoms. Biomarkers nor stratifying risk were used for determining specific follow-up. Data were collected retrospectively from the HFU records.

The post-intervention period was defined as the 12 months after intervention implementation. Patient management during this period of time was according with the personalized protocol based on biomarkers risk stratification. Data were collected prospectively during the 12 months following the intervention implementation. Patients were followed-up by the staff of the Outpatient HF clinic. The follow-up protocol included at the time of the visit a medical examination, completion of a patient questionnaire including all relevant clinical variables, signs and symptoms, medication, NYHA score, use of cardiac resynchronization therapy [CRT] and devices such as implantable cardioverter defibrillators [ICD]), blood testing, electrocardiogram (EKG or ECG), and drug treatment adjustments. Patients that did not complete the 12 months of post-intervention follow-up (dropout or death) were removed from the study.

### Intervention

We designed a specific intervention based on a personalized follow-up protocol according to a risk stratification score that included biomarker levels [NT-proBNP and high sensitivity T-Troponin (hs-TnT)] as first line for risk asses monitoring. According with their specific risk, cardiologist from the HFU categorized the patients into 3 different groups (low, medium and high risk) and specific program of follow-up was created for each group. The risk of hospital admission or HF event was calculated using the Barcelona Bio-Heart Failure Risk Calculator (BCN Bio-HF Calculator) [16, 17]. BCN Bio-HF Calculator provides good accuracy (AUC 0.83) of the individual risk of hospitalization and death yearly and up to 5 years in chronic HF patients. In addition, it incorporates conventional predictor factors as well as cardiac biomarkers that are highly accurate for cardiac malfunction. The calculator was developed with different models allowing its use with different biomarkers. In our study, we used the model that incorporated two biomarkers, NT-proBNP and hs-TnT.

According to risk stratification, the HFU developed a personalized follow-up protocol for each group. Quartiles of the total distribution were selected as cut-off points for the different groups, with follow-up at the HFU after discharge as follows:

- Low-risk patients (score < 5%), follow-up at 90 days and 12 months.
- Medium-risk patients (score 5–15%), follow-up at 60 days and 6 and 12 months.
- High-risk patients (score > 15%), follow-up in 30 days and 3 and 12 months.

### Clinical outcomes

The study compared the differences between the pre- and post-intervention periods to determine the impact in the outcomes. The primary outcome was the readmission rates. The readmission rate was defined as a percentage of patients which were unplanned admitted at the acute care hospitalization unit due to an HF event during the follow up. According with the literature, the rate of hospitalization was calculated at 30 days, 6 months and 1 year of follow up. The absolute number of hospital admissions and the length of stay were also measured.

The secondary outcome of interest was ED visit frequency during the pre- and post-intervention period. Improvement on patient’s quality of life assessed by the NHYHA score during the pre- and post-intervention period was also analyzed.

### Cost analysis

The cost analysis was conducted from the perspective of the Spanish healthcare system, including categories of costs shown in Table 1. All costs were calculated by multiplying the unit cost for the resource used. The average cost of hospitalization was estimated based on the cost established by the Heart Failure diagnosis-related group (DRG) in the Spanish National Health Service and adjusted according to the patient NYHA class. The primary care visits unit costs of €78.45 was calculated according to methods used by Merino et al. [18]. The cost of ED visits was calculated according to the unit cost to the specific DRG of €392.03. The medication unit cost was calculated using the Spanish healthcare prices for Huelva University Hospital in 2018, multiplying by the dose for each patient and calculating the mean in a 1-year period. Laboratory unit costs were calculated as an incremental cost of €14 associated with the cost of the biomarker used during the post-intervention period.

**Table 1:** Categories of costs included in the analysis, per unit

	Cost (€)
Hospitalization cost	3981.89
NYHA 1	2900.76
NYHA 2	3654.64
NYHA 3	4426.22
NYHA 4	6662.33
Primary care visits	78.45
Emergency Department visits	392.03
Heart Failure Unit visits	97.83
Medication cost	1.32
ACE I	0.09
ARA II	0.54
BB	0.04
MRA	0.04
Ivabradine	0.36
Diuretics	0.03
Statins	0.13
Angiotensin receptor-neprilysin inhibitors	0.54
Biomarkers cost	14.00
NT-proBNP (pg/mL)	12.00
hs T-Troponin (ng/mL)	2.00

*ACEI* angiotensin-converting enzyme inhibitor, *ARA* aldosterone receptor antagonist, *BB* betablockers, *MRA* mineralocorticoid receptor antagonist, *NYHA* New York Heart Association, *NT-proBNP* N-terminal pro-brain natriuretic peptide

Quality adjusted life-years (QALYs) were calculated by NYHA class‐specific utility over time multiplied by life years (study period). Thus, utility according to functional classification was as follows: NYHA class I, 0.93 (0.91; 0.96); NYHA class II, 0.78 (0.72; 0.84); NYHA class III, 0.61 (0.59; 0.63); and NYHA class IV, 0.44 (0.42; 0.46) [19, 20]. The total QALYs were calculated as the sum of each functional class multiplied by the number of patients in that class. All costs were adjusted to reflect cost related to the year 2018 and excluding indirect costs. For the cost analysis, we considered a temporary analysis of 1 year. To test uncertainty, we used a non-parametric bootstrap method using the original un-transformed data set to generate an empirical distribution for the difference in mean costs, from which we can obtain the confidence interval around the sample mean estimated for costs. The 95% confidence interval for the mean cost in the two groups of patients was obtained non-parametrically using the 5th and 95th percentiles from the distributions. We also conducted sensitivity analyses by subgroups. After determining the dominant strategy, we calculated the overall budget impact of using that. This estimation was weighed by the number of HF diagnostic cases in one year. To assess the budget impact, we simulated three different scenarios: the best case scenario involved 100% of the HF cases were managed using the new approach, the intermediate-case scenario involved 75% of the HF cases and the worst-case scenario where only 50% of the HF cases were managed according with the new approach.

### Statistical analysis

Sample size was determined based on detecting a difference between groups at 12 months with a power of 80% and a significance level of 5%, detected using a two-tailed t-test, and assuming a loss to follow-up rate of 25%. The rationale for this was based on data from previous studies related to the primary outcome. The sample size required was 143 patients in total, increased to 192 patients due to the expected dropout rate of 25%.

Distributions were examined using the Shapiro–Wilk test to ensure proper statistical evaluation. Continuous variables were expressed as the median with Interquartile Range (IQR) [p25–p75] except glomerular filtration, which was expressed as mean ± standard deviation (SD), and the categorical variables, which were expressed as a frequency (percentage, %) of the population. The differences among the categorical variables were analyzed using the chi-square test (χ^2^), while the Kruskal–Wallis test was used to analyze the differences between independent continuous variables, except glomerular filtration. According with their risk stratification, patients were classified in subgroups, low risk score < 5%, medium risk score 5–15%, and high risk score > 15%). Subgroups analysis was performed for the outcomes and care associate costs. Significance levels less than 5% were considered significant. Statistical analyses of the data were performed using IBM SPSS software (version 22, SPSS Inc., USA).

## Results

### Study cohort

Of the 232 patients initially enrolled 40 patients did not conclude the study. 30 patients did not conclude the proposed follow up protocol and 10 patients died during the follow-up. At the time of the analysis, 192 patients had been included in the study (Fig. 1). Table 2 shows the baseline characteristics of the study cohort. Overall, 79.7% of all patients were male, and the mean age (± SD) was 64 ± 12 years. The duration of the HF was 3 years [2–5]. Common comorbidities included hypertension (69.3%), diabetes (37.5%), chronic renal failure (25.8%), chronic obstructive lung disease (20.3%), and atrial fibrillation (34.9%). Of those with heart disease, 50% had coronary artery disease, although a majority of patients (69.8%) had no HF hospitalizations in the year before enrollment. Most patients, 83.7% were assessed as NYHA Class I or II, reflecting prevalently a mildly symptomatic HF cohort (NYHA I is 36.1% and NYHA II is 47.6%). We found levels of NT-proBNP of 984 [393–2334] pg/mL, and hs-TnT levels of 15 [8–27] ng/mL. We found HF hospitalization readmission rates of 30.2% and 21.9% visited the ED during the 12 months prior to the intervention. The calculate risk for the study groups are showed in the supplementary Table 1.

**Table 2:** Baseline characteristics of the study cohort, for associations between variables depending on the score groups

	Total (N = 192)	Low-risk (n_=_117)	Medium-risk (n = 63)	High-risk (n = 12)	*p* value
Age (years)	65 [57–73]	60 [53–69]	72 [66–77]	73 [65–81]	< 0.001
Gender (female)	20.3	23.9	14.3	16.7	0.292
Arterial hypertension	69.3	58.1	85.7	91.7	< 0.001
Dyslipidemia	64.1	52.1	82.5	83.3	< 0.001
Diabetes mellitus	37.5	25.6	54.0	66.7	< 0.001
COPD	20.3	13.7	30.2	33.3	0.016
Chronic renal failure	25.8	13.0	42.9	58.3	< 0.001
Previous atrial fibrillation	34.9	22.2	50.8	75.0	< 0.001
LVEF	30 [27–36]	30 [28–36]	30 [28–36]	27 [25–32]	0.116
Ischemic etiology	50	40.2	65.1	66.7	0.003
Duration of HF (years)	3 [2–5	2 [2–4]	5 [3–7]	6 [3–9]	< 0.001
Functional class					
NYHA 1	36.1	44.8	23.8	16.7	
NYHA 2	47.6	46.6	52.4	33.3	< 0.001
NYHA 3	16.2	8.6	23.8	50.0	
ICD/CRT	12.0	5.1	20.6	33.3	0.001
Heart rate	61 [55–70]	60 [55–66]	63 [60–70]	64 [59–80]	0.107
NT-proBNP (pg/mL)	984 [393–2334]	599 [244–1211]	2045 [860–3664]	3494 [1503–8541]	< 0.001
hs T-Troponin (ng/mL)	15 [8–27]	11 [6–16]	24 [17–40]	53 [39–68]	< 0.001
Glomerular filtration (mL/min/1.73 m^2^)	76.07 ± 27.75	86.36 ± 23.66	62.54 ± 22.59	46.75 ± 38.50	< 0.001
Sodium (mEq/L)	141 [140–143]	141 [140–143]	142 [141–144]	139 [138–141]	0.005
ACEI/ARB	60.4	62.4	58.7	50.0	0.667
ARNI	38.0	35.9	39.7	50.0	0.598
Betablockers	95.8	97.4	95.2	83.3	0.064
MRA	78.1	76.1	82.5	75.0	0.584
Diuretics	67.7	47.0	100.0	100.0	< 0.001

Data are presented as median and Interquartile Range [p25–p75] for continuous variables and percentages for categorical variables

*ACEI* angiotensin-converting enzyme inhibitor, *ARB* angiotensin receptor blocker, *ARNI* angiotensin receptor-neprilysin inhibitor, *COPD* chronic obstructive pulmonary disease, *CRT* cardiac resynchronization therapy, *ICT* implantable cardioverter defibrillators, *LVEF* left ventricular ejection fraction, *HF* heart failure, *MRA* mineralocorticoid receptor antagonist, *NYHA* New York Heart Association, *NT-proBNP* N-terminal pro-brain natriuretic peptide

The subgroup analysis showed that the patients in the highest risk group were more likely to be older, had more comorbidities, and their heart disease was at a more advanced stage. We found levels of NT-proBNP of 599 [244–1211] pg/mL, 2045 [860–3664] pg/mL and 3494 [1503–8541] pg/mL (*p* < 0.001) and hs-TnT levels of 11 [6–16] pg/mL, 24 [17–40] pg/mL and 53 [39–68] pg/mL (*p* < 0.001) for the low, medium and high risk patients respectively. Improvement in NT-proBNP levels were found after 12 months of follow-up in the post-intervention period, 392 [192–949] pg/mL, 1923 [800–3685] pg/mL and 2283 [1263–4409] pg/mL for the low, medium and high risk patients respectively.

For the low risk group 91.4% of the patients were in the lowest functional class (NYHA class I or II), 76.5% for the medium risk group and 50% for the high-risk groups. No differences between groups were found in the LVEF or in the use of therapies included Angiotensin-receptor-neprilysin-inhibitor (ARNI), Angiotensin Converting Enzyme Inhibitor (ACEI)/Angiotensin Receptor Blocker (ARB) or beta-blockers (BB).

### Clinical outcomes

*Primary outcomes* Table 3 compares the main outcome of rate of admission 30 days, 6 months and 12 months between pre- and post-intervention periods, analyzing patients by risk groups. Of the total, 7.8% had been admitted at least once in the 30 days prior to the baseline visit, which reduced to 1% in the 30 days following the intervention (*p* = 0.002). The respective reductions being admitted in the pre-intervention and post-intervention periods, in the low-, medium- and high-risk groups were 5.1% (from 5.1 to 0% (*p* = NA), 7.9% (from 9.5 to 1.6%, (*p* = 0.125), and 16.7% (from 25 to 8.3% (*p* = 0.625). Overall, a significant reduction (14%) was observed when comparing 6 months in the pre-intervention period (20.3%) and 6 months post-intervention (6.3%) (*p* < 0.001). Of the 192 patients, 30.2% of the sample had been admitted at least once in the pre-intervention period; and in the post-intervention period, this number decreased to 10.4% in a year (*p* < 0.001). The respective reductions in the low-, medium- and high-risk groups before and after the intervention were: 17.1% (from 22.2 to 5.1% (*p* < 0.001); 23.8 (from 38.1 to 14.3% (*p* = 0.125); and 25% (from 66.7 to 41.7% (*p* = 0.453).

**Table 3:** Rehospitalization rates 30 days, 6 months and 12 months for the pre-intervention and post-intervention periods

Sample size	Pre-intervention	Post-intervention	*p* value
30-days admissions (%)			
All (N = 192)	7.8	1	0.002
Low risk (n = 117)	5.1	0	NA
Medium risk (n = 63)	9.5	1.6	0.125
High risk (n = 12)	25	8.3	0.625
6-months admissions (%)			
All (N = 192)	20.3	6.3	< 0.001
Low risk (n = 117)	15.4	1.7	< 0.001
Medium risk (n = 63)	23.8	11.1	0.096
High risk (n = 12)	50	25	0.453
12-months admissions (%)			
All (N = 192)	30.2	10.4	< 0.001
Low risk (n = 117)	22.2	5.1	< 0.001
Medium risk (n = 63)	38.1	14.3	0.003
High risk (n = 12)	66.7	41.7	0.453

When we consider the absolute data, in the cohort of 192 patients, we found a significant reduction (19.8%) in the number of patients admitted during the post-intervention period (20 patients; 10.4%) compared with the pre-intervention period (58 patients; 30.2%) (*p* < 0.001). The difference was significant for the low, medium and high-risk groups. We also found a significant reduction of 32 in the number of hospital admissions (from 78 to 46 admissions) (*p* < 0.001) and in the hospital length of stay of 7 days (from 15 to 9 days) (Additional file 1: Table S2).

*Secondary outcomes* The rate of visits in the ED also decreased in the post-intervention period (Table 4). In the 12 months before the study, the number of visits was 64, which decreased to 20 after the intervention (*p* < 0.001). A marked functional improvement was observed in the post-intervention period (Table 5). In total, 31.1% of the patients improved at least one class in NYHA score, 61.6% remained the same, and 7.3% got worse. The number of asymptomatic patients also increased by 10%.

**Table 4:** Change in ED visits pre-intervention and post-intervention periods (total and by subgroups)

	Pre-intervention	Post-intervention	*p* value
30-day ED visits (%)			
Total (N = 192)	4.7	0	NA
Low risk group (n = 117)	1.7	0	NA
Medium risk group (n = 63)	7.9	0	NA
High risk group (n = 12)	16.7	0	NA
6-months ED visits (%)			
Total (N = 192)	12.5	2.1	< 0.001
Low risk group (n = 117)	7.7	0.9	0.021
Medium risk group (n = 63)	15.9	1.6	0.012
High risk group (n = 12)	41.7	16.7	0.375
12-months ED visits (%)			
Total (N = 192)	21.9	7.3	< 0.001
Low risk group (n = 117)	12.8	3.4	0.013
Medium risk group (n = 63)	31.7	9.5	0.007
High risk group (n = 12)	58.3	33.3	0.453

**Table 5:** Change in functional class (NYHA) pre- and post-intervention (total and by subgroups)

	Improved (%, 95% CI)	No change (%, 95% CI)	Worse (%, 95% CI)
Total	31.07 (24.71, 37.78)	61.58 (54.94%, 67.86)	7.34 (3.87, 11.3)
Low	28.70 (20.72, 37.28)	65.74 (56.91, 73.69)	5.56 (1.9, 10.48)
Medium	37.93 (26.15, 50)	53.45 (41.43, 65.08)	8.62 (1.75, 16.67)
High	22.22% (0, 55.6)	55.56 (20, 90)	22.22 (0, 50)

Confidence intervals were calculated with n = 1000 and 95% confidence level

NYHA changes were calculated comparing the NYHA class at the end of the pre-intervention period and NYHA class at the end of the post-intervention period

### Costs analysis

**Fig. 2:**
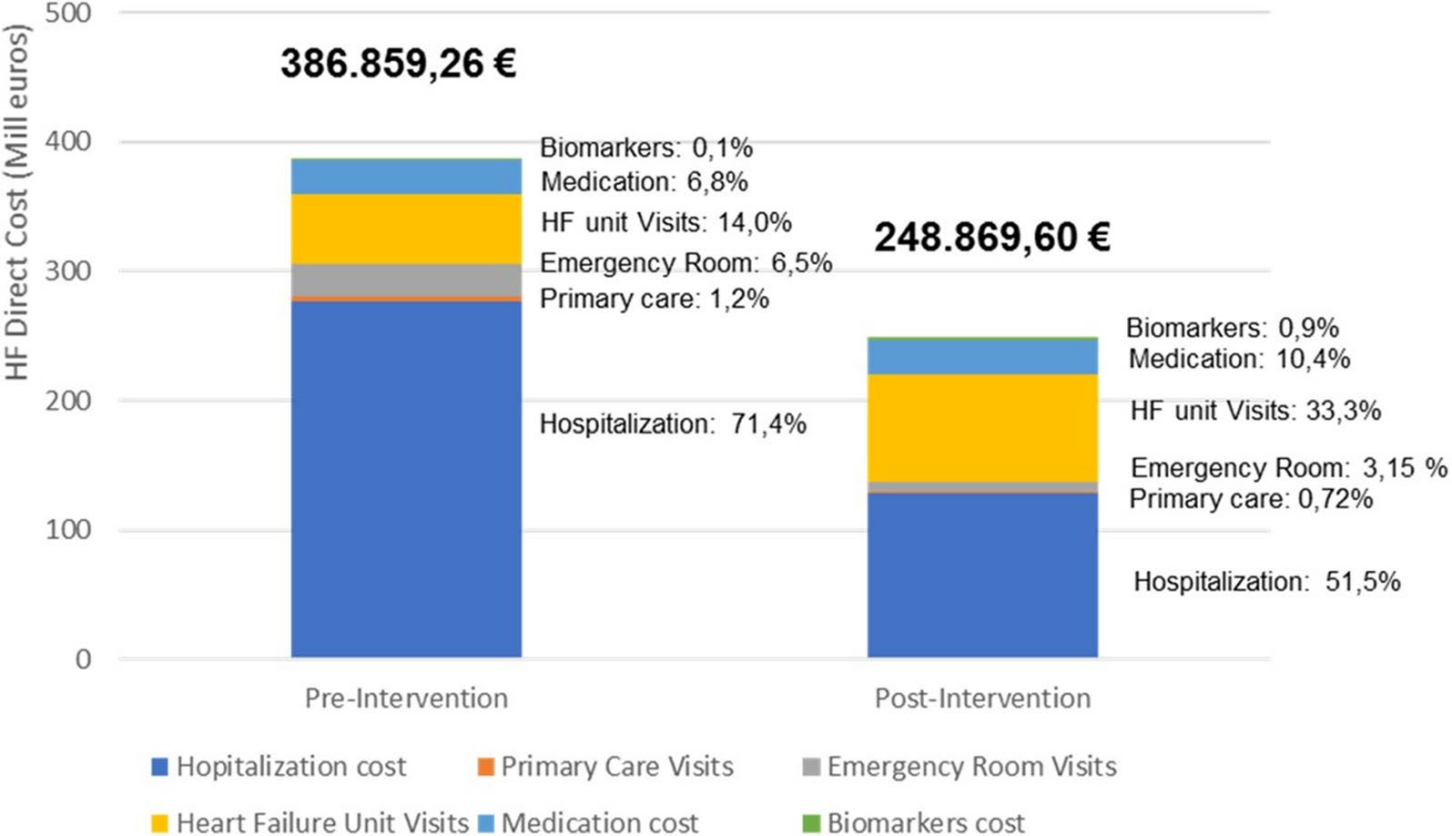
HF cost according to groups. Values are expressed in euros

Table 6 compares the total care associated cost and the specific components during the follow-up between the groups. The overall cost per patients of applying the new follow-up intervention was € 72,769 lower compared with standard care pre-intervention (from € 201,189 to € 128,420 per patients). We found a significant cost reduction in most of the categories considered. The most important cost reduction was related to costs associated with hospitalization, demonstrating a significant reduction of € 771.1 per patient (from € 1438.75 to € 667.65 per patient) (*p* < 0.05) (Fig. 2). The subgroups analysis showed a significant cost reduction for the most cost categories for the low, medium and high risk groups (Additional file 1: Table S3).

**Table 6:** Differences in costs per patient during the 12 months of follow-up

Cost categories	Pre-intervention (€, 95% CI)		Post-intervention (€, 95% CI)		Difference	
Hospitalization cost	1438.75	(1025.70,1961.32)	667.64	(316.53, 1133.74)	− 771.11	(− 1234.81, − 541.77)
primary care visits	23.69	(16.33, 32.68)	9.39	(4.90, 15.93)	− 14.3	(− 24.92, − 5.30)
Emergency department visits	130.67	(86.69, 191.97)	40.83	(22.45, 63.29)	− 89.84	(− 155.17, − 38.73)
Heart failure unit visits	282.26	(271.45, 291.57)	431.05	(416.73, 447.67)	148.79	(123.33, 173.82)
Medication cost	136.49	(123.42, 150.33)	135.25	(123.03, 148.17)	− 1.24	(− 9.94, 6.95)
Total	2011.86	(1581.16, 2540.90)	1284.19	(924.83, 1764.79)	− 727.7	(− 1166.66, − 333.11)

Differences are showed as post-intervention costs less pre-intervention costs. Cost savings shown with a negative difference)

We found a significates reduction of the cost associate to emergency department visits of € 89.84 per patients (from € 130.68 to € 40.84 per patients), and there was a significant reduction in the costs associated with primary care visits and with medication. There was a corresponding incremental cost related to the use of biomarkers (€ 9 per patients) and HFU visits (€ 148.79 per patients).

**Fig. 3:**
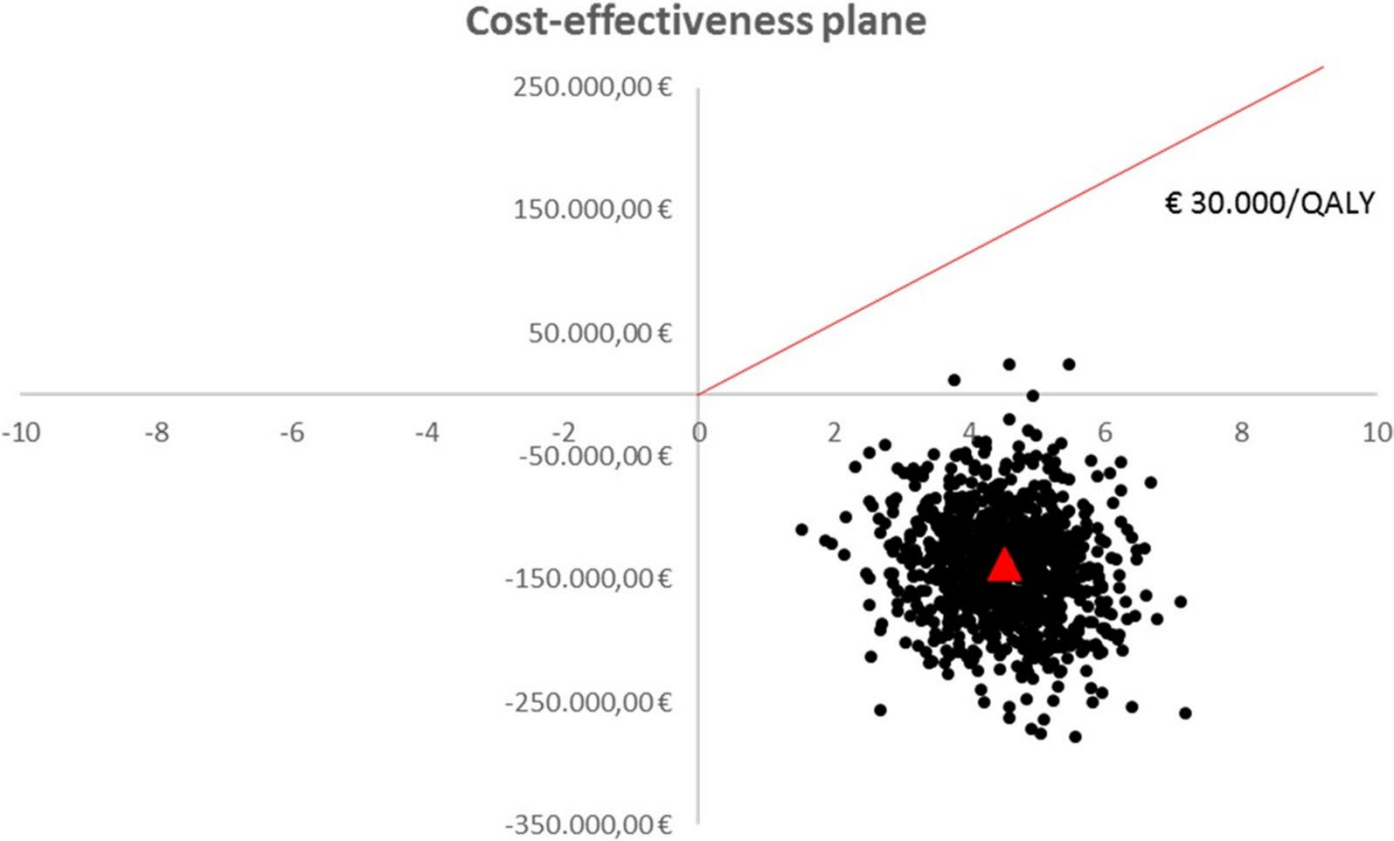
One thousand bootstrap estimates of the differences in mean cost and mean (quality adjusted) survival time between pre- and post-intervention. The cost-effectiveness plane represents the incremental costs and effects of the intervention compared to regular care. The origin represents the comparator treatment, in this case non personalized follow up management. If the new intervention lies to the right of the origin it is more effective (i.e., it has more QALYs than those with regular care), or if it lies to the left of the origin it is less effective. If it lies above the x-axis then it is more costly than B, and vice versa

Utilizing the personalized biomarker approach produced a total of 113.6 QALYs (95% CI 108.5 to 118.2) compared with 109.1 QALYs (95% CI 104.2 to 113.4) for regular care, an increment of 4.5 QALYs (95% CI 2.9 to 6.1). The new approach was dominant (both less costly and more effective). The sensitivity analysis indicates that the new approach is the most cost-effective decision (Fig. 3).

The budget impact analysis showed a potential saving between € − 704.03 patient-years (p-y) (95% CI 1141.65 to − 273.83) when the savings per patient was translated to the overall patient population in the best-case scenario (100% of the HF patients conducted using our new approach), to € 352.01 p-y (95% CI − 570.83 to − 136.91) in the worst-case scenario (100% of the HF patients conducted using our new approach), with a medium-case scenario (50% of the HF patients conducted using our new approach) with a potential savings of € 528.02 p-y (95% CI − 856.24 to − 205.37). Based on the 80,000 hospital admissions for HF that occur every year in Spain [18], the budget impact, considering only direct costs, could be between €-56,322,308 (95% CI − 91,332,342 to − 21,906,326) in the best-case scenario and € − 28,161,154 (95% CI − 45,666,171 to − 10,953,163) in the worst-case scenario.

## Discussion

Although a principal goal of HF management is to improve patient outcomes and quality of life, few studies have evaluated the possibility of carrying out personalized management to improve them, as we did. The primary finding of this study is that a strategy of personalized follow-up based on cardiac biomarkers for patients with chronic HFrEF was more effective than regular care in reducing the composite outcome of readmission rates. Significantly different results were seen in other clinical outcomes, including a reduction of ED visits and improvement in patients’ quality life assessed by NYHA classification during the monitoring period. There was also a significant reduction in the HF-associated cost using the personalized approach compared with the strategy used in regular care.

A number of biomarkers are now well established to be a prognostic value in HFrEF [15]. The combination of biomarkers with traditional risk factors to refine risk prediction, identify patients at-risk and intensify their management, may provide an approach to improve HFrEF management [21]. Our study proposed an individualized management based on a clinical model of risk stratification supported by biomarkers (NT-proBNP and hs-TnT) whose results in admission rates, are comparable with the published literature; the 1-year incidence rate of readmission has been reported at 14.5% among 12,440 chronic HF patients from different geographical areas [22]. A systematic review of different strategies for HF management found 6/19 trials demonstrated statistically significant reductions in HF readmissions with multidisciplinary management and personalized follow-up strategies [23]. The average reduction in readmission rates was 12.37% over 12 months’ follow-up, varying between 2.71 and 17.81% [23, 24]; differences between types of interventions were not found. Although difficult to compare across studies, the reduction in hospitalization readmissions after 1 year of follow-up was higher in this study (17–25% across risk groups). This may result from the focusing of resources on those patients at highest risk. In addition, our study proposes a personalized follow-up procedure based on the patient’s risk, assessed in everyday clinical scenario, and suggests that greater attention should be given to the high-risk cohort to minimize the risk of readmissions.

As many as 77% of high-risk patients initially present to the ED [25], and close follow-up after discharge has been shown to decrease ED admissions [26, 27]. Our results differ from these studies and show a significant reduction of 68.7% in ED admissions during the follow-up period. Although the majority of hospitalizations for HF begin in the ED, close outpatient follow-up and management has been proposed as a viable strategy to reduce readmissions.

Our study showed a significant improvement in patient’s quality of life, symptoms and functional status demonstrated by the reduction in the percentage of patients in NYHA class III and an increasing number of patients in NYHA class I and II at follow-up. Those results are aligning with previous studies, Romano et al. [28] and other authors found a significant improvement in NYHA class after different interventions in patients with HFrEF [29]. The NYHA classification is the most commonly used system to describe the impact of heart failure on a patient's daily activities [30] and is recommended in all guidelines [10, 15] as a useful tool to assess the functional limitations due to HF. It has been shown to be an important predictor of outcomes in heart failure [31], and NYHA functional class was the most dominant predictor, among several somatic variables, associated with a decrease in quality of life [32]. Also, a relationship between the NYHA functional class has been demonstrated with various life questionnaires in patients with HF [33].

The results of the current study strongly support the current guidelines regarding NYHA class reduction as primary endpoint for therapeutically interventions in HF.

Our cost analysis results agree with those proposed by other groups; Lesyuk et al. [34] found that 44–96% of the direct costs of HF care are due to hospitalization, suggesting that reduction of readmission rates would reduce the direct cost associated with HF. Our study also reports a significant reduction in the cost related to emergency admission and primary care visits, which we believe are associated with better control of the patients after the intervention. The significant reduction in NYHA class could also contribute to the cost reduction; patients with NYHA IV have between 8 and 30 times higher healthcare costs than patients with NYHA II [35, 36].

Data for the cost analysis of biomarker-guided personalized outpatient management of HF patients are limited. Our cost analysis showed that personalized follow-up was the dominant approach, with a potential saving of €− 704,028.85 per 1000 p-y. Given the expected cost differential between serial biomarker monitoring and hospitalization for HF, even a modest reduction in admissions due to biomarker personalized follow-up could result in net cost savings. Biomarker personalized therapy has a high probability of being cost-effective in HF patients with reduced LVEF [35].

This study focuses on a personalized follow-up based on cardiac biomarkers in everyday clinical practice and in an uncontrolled sample of patients with chronic HFrEF. In addition to previous studies, ours not just only evaluated the classic endpoint used in clinical trial but also achieved the results in patient’s outcomes and care associated cost improvement. Along with the vague recommendations proposed in the literature, we proposed a standard strategy for patient management which is easy to use and helpful for clinicians less well versed in the guidelines who may be less familiar with recognizing and treating HFrEF. Contemporary data suggest that glaring gaps in care quality exist for those affected by HFrEF, who are not very often managed according with their specific risk. Thus, a tool to optimize HFrEF management allowing the recognition of patients at higher risk and real-time personalized follow-up, is sought. Our study achieves some of the most important pivotal issues pointed by the groups of expert of the American Associated of Cardiologist that remain unresolved in the literature in relation with the management of the HFrEF [10]. Our study could constitute a good starting point to further research to clarify the concerns about the best management for patients with HFrEF.

Many limitations exist in the current study beginning with our study design which was based on an uncontrolled before and after study which has limitations. The readers should take in consideration when they interpret the result showed. These results should be validated in other studies with rigorous methodology evolved for prospective, randomized, controlled clinical trials with larger sample size. Thus, the model of care for HF is currently carried out according to local practices, only reflects the experience of a single hospital, and the analyses were conducted from the Spanish health system perspective, including pricing. Hospitalization cost was estimated based on the cost established by the Heart Failure DRG, no individual cost associated to procedures or cardiovascular tests were included individually. However, general population data ranges were used in the sensitivity analyses to improve generalizability. Demonstrating the prices and efficacy necessary for cost analysis at each threshold makes our results relevant to other systems, and transferrable to clinical practice. Finally, the assessment of quality life status using the NYHA classification, although it is a standard practice in HF, is highly subjective and shows the perspective of the clinicians as opposed of the patients, limiting its usefulness. Those results should be validated used dedicated stronger instrument such as Kansas City Cardiomyopathy questionnaire or Minnesota Living with Heart Failure Questionnaire.

## Conclusions

In conclusion**,** personalized handling in HF, with novel clinical strategies for optimizing treatment, improving outcomes, and reducing the cost in HF, is sorely needed. Our strategy of personalized follow-up based on cardiac biomarkers to optimize HF management, represents a good new approach to achieve these goals and it should be seen as a starting point for further studies focusing on improving management of HFrEF.

## Supplementary Information

**Additional file 1.** **Supplementary table 1:** Risk of Heart Failure admission for the pre- and post-intervention period. **Supplementary table 2:** Comparison of the number the patients admitted, number of hospital admissions and length of stay between the pre- and post-intervention period. **Supplementary table 3:** Differences in total costs during the 12 months of follow-up for the pre-intervention and post-intervention groups by subgroups categories.

## Data Availability

The datasets used and/or analyzed during the current study are available from the corresponding author on reasonable request.

## Abbreviations

HF: Heart failure
HFrEF: Heart failure with reduced-ejection-fraction
ED: Emergency department
NYHA: New York Heart Association
HFU: Heart Failure Unit
LVEF: Left ventricular ejection fraction
CRT: Cardiac resynchronization therapy
ICD: Implantable cardioverter defibrillator
ECK/ECG: Electrocardiogram
NT-proBNP: N-terminal pro b-type natriuretic peptide
Hs-TnT: High Sensitivity Troponin T
BCN Bio-HF: Calculator Barcelona Bio-Heart Failure Risk Calculator
AUC: Area under the ROC curve
DRG: Diagnosis-related group
QALYs: Quality adjusted life-years
IQR: Interquartile range
SD: Standard deviation
ARNI: Angiotensin-receptor-neprilysin-inhibitor
ACIE: Angiotensin converting enzyme inhibitor
ARB: Angiotensin receptor blocker
BB: Beta-blockers
