# Supplementary material for: Biomarkers-based personalized follow-up in chronic heart failure improves patient’s outcomes and reduces care associate cost
Source: Health Qual Life Outcomes. 2021 May 8;19:142. doi: 10.1186/s12955-021-01779-9 (PMC8106851; doi:10.1186/s12955-021-01779-9)
Supplement: Supplementary file 1 — Additional file 1. Supplementary table 1: Risk of Heart Failure admission for the pre- and post-intervention period. Supplementary table 2: Comparison of the number the patients admitted, number of hospital admissions and length of stay between the pre- and post-intervention period. Supplementary table 3: Differences in total costs during the 12 months of follow-up for the pre-intervention and post-intervention groups by subgroups categories. [file 12955_2021_1779_MOESM1_ESM.docx]

Supplementary table 1: Risk of Heart Failure admission for the pre- and post-intervention period

|  | Total  (n=192) | Low-risk  (n=117) | Medium-risk  (n=63) | High-risk  (n=12) | **p value** |
| --- | --- | --- | --- | --- | --- |
| pre-intervention | 4.25 [0.30-8.55] | 0.50 [0.20-3.90] | 9.60 [6.70-12.80] | 21.70 [17.55-26.78] | **<0.001** |
| post-intervention | 3.00 [0.20-5.95] | 0.30 [0.20-3.65] | 5.50 [3.10-7.40] | 10.00 [4.10-13.88] | **<0.001** |
| **p value** | **<0.001** | **0.579** | **<0.001** | **0.003** |  |

Results are shown in median and IQR.

Supplementary table 2: Comparison of the number the patients admitted, number of hospital admissions and length of stay between the pre- and post-intervention period

|  |  | Total | Low-risk | Medium-risk | High-risk | p value |
| --- | --- | --- | --- | --- | --- | --- |
|  |  | (n=192) | (n=117) | (n=63) | (n=12) |  |
| **Number of patients admitted** | **pre-intervention** | 58 (30,2%) | 26 (22,2%) | 24 (38,1%) | 8 (66,7%) | 0.003 |
|  | **post-intervention** | 20 (10,4%) | 6 (5,1%) | 9 (14,3%) | 5 (41,7%) | <0.001 |
|  | **p value** | <0.001 | <0.001 | 0.003 | 0.453 |  |
| **Number of Hospital Admissions** | **pre-intervention** | 78 | 29 | 28 | 21 | <0.001 |
|  | **post-intervention** | 46 | 15 | 20 | 11 | <0.001 |
|  | **p value** | <0.001 | <0.001 | 0.055 | 0.232 |  |
| **Length of stay** | **pre-intervention** | 15 [6-29] | 15 [11-26] | 10 [4-16] | 29 [25-49] | 0.519 |
|  | **post-intervention** | 9 [2-21] | 8 [5-9] | 10 [2-18] | 21 [11-41] | 0.214 |
|  | **p value** | 0.126 | 0.109 | 0.789 | 0.180 |  |

| **Cost Categories** | **Pre-intervention** | **Post-intervention** | **Difference** |
| --- | --- | --- | --- |
| **Hospitalization cost (€)** | 276,240.59 | 128,188.07 | -148,052.52 |
| High Risk (€) | 75,561.69 | 21,317.72 | -54,243.97 |
| Medium Risk (€) | 97,105.75 | 56,350.43 | -40,755.31 |
| Low Risk (€) | 103,573.15 | 50,519.92 | -53,053.22 |
| **Primary Care Visits (€)** | 4,550.10 | 1,804.35 | -2,745.75 |
| High Risk (€) | 549,15 | 0 | -549.15 |
| Medium Risk (€) | 2,588.85 | 784.5 | -1,804.35 |
| Low Risk (€) | 1,412.1 | 1,019.85 | -392.25 |
| **Emergency Department Visits (€)** | 25,089.92 | 7,840.60 | -17,249.32 |
| High Risk (€) | 4,312,33 | 0 | -4,312,33 |
| Medium Risk (€) | 14,505,11 | 4,312.33 | -10,192,78 |
| Low Risk (€) | 6,272,48 | 3,528.27 | -2,744,21 |
| **Heart Failure Unit Visits (€)** | 54,195.65 | 82,762.83 | 28,567.18 |
| High Risk (€) | 3,555.33 | 6,498,03 | 2,942.7 |
| Medium Risk (€) | 33,440.21 | 47,296 | 13,855.79 |
| Low Risk (€) | 17,200.11 | 28,968.8 | 11,768.69 |
| **Medication cost (€)** | 26,207.00 | 25,969.75 | -237.25 |
| High Risk (€) | 1,328.6 | 1,405.25 | 76.65 |
| Medium Risk (€) | 8,924.25 | 7,604.775 | -219 |
| Low Risk (€) | 15,954.15 | 16,959.725 | 2,106.05 |
| **Total (€)** | 386,283.26 | 246,565.60 | -139,717.65 |
| High Risk (€) | 85,307.106 | 29,221 | -56,086.10 |
| Medium Risk (€) | 156,564.16 | 11,6348.03 | -40,216.13 |
| Low Risk (€) | 144,411.98 | 10,0996.56 | -43,415.42 |

Supplementary table 3: Differences in total costs during the 12 months of follow-up for the pre-intervention and post-intervention groups by subgroups categories
